# Extramedullary blast crisis of chronic myelogenous leukemia as an initial presentation^☆^

**DOI:** 10.1016/j.lrr.2013.07.002

**Published:** 2013-08-13

**Authors:** Shokichi Tsukamoto, Satoshi Ota, Chikako Ohwada, Yusuke Takeda, Masahiro Takeuchi, Emiko Sakaida, Naomi Shimizu, Koutaro Yokote, Tohru Iseki, Chiaki Nakaseko

**Affiliations:** a Department of Hematology, Chiba University Hospital, 1-8-1 Inohana, Chuo-ku, Chiba 260-8670, Japan; b Department of Pathology, Chiba University Hospital, Chiba, Japan; c Division of Transfusion Medicine and Cell Therapy, Chiba University Hospital, Chiba, Japan; d Department of Clinical Cell Biology and Medicine, Chiba University Graduate School of Medicine, Chiba, Japan

**Keywords:** Extramedullary blast crisis, Chronic myelogenous leukemia (CML), Dasatinib

## Abstract

Extramedullary blast crisis of chronic myelogenous leukemia (CML) is defined as the development of extramedullary disease caused by the infiltration of blasts regardless of proliferation of blasts in the bone marrow. The onset of extramedullary blast crisis in the newly diagnosed patients is known to be extremely rare. Here, we present a case of extramedullary blast crisis of CML as an initial presentation in a 17-year-old female presenting with pain in the left femur tumor. This case was treated successfully with dasatinib and allogeneic hematopoietic stem cell transplantation, with achievement of long-term remission.

## 1 Case report

**Fig. 1:**
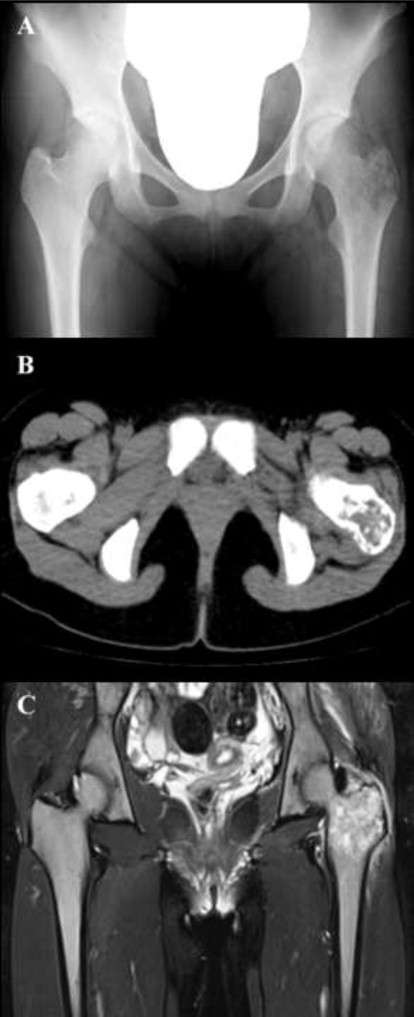
Radiography, CT, and MRI findings at the development of extramedullary blast crisis of CML. (A) Radiography revealed a radiolucent region, and (B) CT and (C) MRI revealed an osteolytic tumor at the greater trochanter of the left femur.

**Fig. 2:**
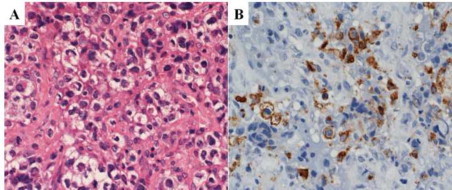
A biopsy specimen of the left femur tumor. (a) Hematoxylin and eosin staining, high-power field. Extramedullary blast proliferation with fibrosis was seen. (b) Immunohistochemical staining for CD68 (KP-1), high-power field. The blast cells were partially positive for CD68 (KP-1).

A 17-year-old female presented with pain in the left hip joint in April 2010. Radiography revealed a radiolucent region at the greater trochanter of the left femur (Fig. 1A). Computed tomography (CT) and magnetic resonance imaging (MRI) revealed an osteolytic tumor at the greater trochanter of the left femur (Fig. 1B and C). Blood analyses showed an elevated white blood cell (WBC) count of 130,200/µL; a decreased hemoglobin level of 9.4 g/dL; and an increased platelet count of 465,000/µL. The differential leukocyte count revealed 3.4% blasts, 0.4% promyelocytes, 9.2% myelocytes, 1.8% metamyelocytes, 53.6% neutrophils, 12.4% eosinophils, and 15.6% basophils. Examination of the bone marrow showed increased cellularity due to granulocytic proliferation with a maturation pattern similar to that observed in peripheral blood, with a blast count of 6.5% and a basophil count of 5.6%. Chromosomal analyses of the bone marrow cells revealed an t(9;22) (q34;q11.2) abnormality. The type of *BCR-ABL* fusion transcripts was major *BCR-ABL* which led to a p210 fusion protein. A biopsy specimen of the left femur tumor revealed extramedullary blast proliferation with fibrosis (Fig. 2a). An immunohistochemical study demonstrated the blast cells were partially positive for CD68 (KP-1) (Fig. 2b), CD68 (PGM-1), and myeloperoxidase, and negative for terminal deoxynucleotidyl transferase (TdT), CD3, CD79a, and CD34. Chromosomal analyses of the tumor cells of the left femur also revealed a t(9;22)(q34;q11.2) abnormality. The diagnosis was extramedullary myeloid blast crisis of chronic myelogenous leukemia (CML).

Initially she was administered imatinib (400 mg once daily). The WBC count in peripheral blood decreased rapidly. She achieved a complete hematologic response within 23 days after the initiation of imatinib therapy. Radiography revealed that the radiolucent region at the greater trochanter of the left femur had also improved. However, the pain in her left hip joint suddenly worsened on the 54th day of imatinib treatment. Radiography and CT showed that the osteolytic tumor at the greater trochanter of the left femur had enlarged. The WBC count of peripheral blood was 3800/µL with normal differential leukocyte count. Examination of the bone marrow showed that the bone marrow remained in a chronic phase with a minor cytogenetic response. Mutation of the *BCR-ABL* kinase domain was not detected. [18F]Fluorodeoxyglucose positron emission tomography (FDG-PET) revealed that abnormal uptake of FDG was limited to the tumor in the left femur. Because of the severe pain and the risk of pathological fracture, she was treated with involved field radiotherapy at 30 Gy. In addition, imatinib was switched to dasatinib (70 mg, twice daily) because the residual tumor was considered to be resistant to imatinib.

Two months from the initiation of dasatinib, abnormal FDG uptake of the left femur tumor had disappeared. Examination of peripheral blood and the bone marrow examination showed that a complete cytogenetic response was achieved and a ratio of *BCR-ABL* to *ABL* on the international scale was 0.21%. Her disease was well controlled with dasatinib. However, we considered that prompt allogeneic hematopoietic stem cell transplantation (allo-HSCT) was necessary for long-term remission. Although she did not have a sibling donor with identical human leukocyte antigens (HLAs), her mother was HLA-1 allele-mismatched for the graft-versus-host direction. Therefore, we performed allo-HSCT from her mother 4 months after the diagnosis. She achieved a complete molecular response with sensitivity of 4.5-log 1 month after transplantation, and the osteolytic region at the greater trochanter of the left femur gradually ossified. Considering the high prevalence of relapse, dasatinib maintenance was started on the 120th day after transplantation. The dose was reduced to 20 mg once daily, but she could not tolerate dasatinib (grade-2 malaise and grade-3 maculopapular rash). She has been in complete remission without the need for a tyrosine kinase inhibitor (TKI) for two and a half years after transplantation, with excellent performance status.

### 1.1 Discussion

Extramedullary blast crisis of CML is defined as the development of extramedullary disease caused by the infiltration of blasts regardless of proliferation of blasts in the bone marrow. Extramedullary blast crisis is, after a few months, almost always followed by hematological blast crisis, so it is considered to be an early sign of blast crisis in the bone marrow [1–3]. The prevalence of extramedullary blast crisis has been reported to be 7–17% in patients with a blast phase (BP) [1,2,4]. From the point of an initial presentation, the onset of extramedullary blast crisis in the newly diagnosed patients is known to be extremely rare because the prevalence of an accelerated phase and BP as initial presentations has been reported to be only 5–10% in CML patients [5].

Extramedullary blast proliferations commonly present in the lymph node, skin, spleen, bone, or central nervous system (CNS), but can occur anywhere, and may consist of myeloid or lymphoid lineages. Other changes (e.g., reticulin or collagen fibrosis, bone remodeling) are more frequently seen in BP [6]. Extramedullary blast proliferation in our patient was accompanied by fibrosis, which helped us to make a diagnosis of BP.

Considering the high prevalence of relapse in patients with BP after allo-HSCT (≤30–40%) post-transplant monitoring and prophylactic/therapeutic strategies are very important. In general, post-transplant maintenance with imatinib is well tolerated, and serious adverse events are resolved successfully after dose reduction [7]. There have been only a few reports of post-transplant dasatinib administration. Those studies suggested that the side effects of dasatinib could be readily managed by dose reduction or treatment interruption [8] and dasatinib could be useful for post-transplant prophylactic/therapeutic strategies for patients with CML or Philadelphia-positive acute lymphoblastic leukemia (especially for extramedullary relapse) because dasatinib can penetrate extramedullary tissue or the CNS [9]. However, algorithms for dosage, intervals from transplantation, and duration of application have not been developed. Our patient could not tolerate dasatinib after transplantation even though the dose was sufficiently reduced compared with that of pre-transplant administration. This finding was not in accordance with the results of previous studies. Although the reason for this difference is not clear, there may be some patients in whom the blood level of dasatinib becomes higher than pre-transplant because of their organ damages after transplantation. The optimal usage of post-transplant dasatinib should be clarified by the clinical trial in the future.

The phenomenal success of therapy with TKIs in CML has drastically changed the prognosis of this disease [10]. In contrast to the pre-imatinib era, patients with BP are much fewer and those with extramedullary blast crisis are even fewer. However, the initial onset of BP will not change in the future; there is still a chance to encounter an extramedullary blast crisis as an initial presentation. The results of this case study will raise awareness and offer suggestions for management of this rare manifestation of CML.
